# Smartphone-Assisted Paper-Based Analytical Device for Rapid Colorimetric Detection of Total Reducing Sugars in Honey

**DOI:** 10.3390/s26103031

**Published:** 2026-05-11

**Authors:** Alicia Carro, Isela Lavilla, Carlos Bendicho, Vanesa Romero

**Affiliations:** Centro de Investigación Mariña, Grupo QA2, Edificio CC Experimentais, Departamento de Química Analítica y Alimentaria, Universidade de Vigo, Campus de Vigo, As Lagoas Marcosende, 36310 Vigo, Spain; alicia.carro.pazos@alumnado.uvigo.gal (A.C.); isela@uvigo.gal (I.L.); bendicho@uvigo.gal (C.B.)

**Keywords:** paper-based analytical device, smartphone-assisted analysis, colorimetric detection, glucose, fructose, honey analysis, food quality control, monosaccharides, reducing sugars

## Abstract

**Highlights:**

**What are the main findings?**

- The study presents a smartphone-assisted paper device capable of rapidly quantifying total reducing sugars, expressed as the sum of glucose and fructose, in honey samples through colorimetric analysis.
- The method demonstrates accurate agreement with certified reference material values and commercial honey label contents, and with results obtained using the official AOAC Lane–Eynon method, validating its analytical reliability.

**What are the implications of the main findings?**

- The device offers a low-cost analytical approach suitable for routine honey quality control in non-specialized or resource-limited laboratories, relying on basic equipment to perform a colorimetric reaction.
- The proposed approach supports broader adoption of accessible digital–analytical tools for food authentication and consumer protection.

**Abstract:**

Determining reducing sugars in honey is key to ensuring its quality and authenticity. Honey mainly contains fructose and glucose, which represent around 90–95% of the total sugar content, and is one of the most adulterated foodstuffs worldwide. Fraudulent practices such as the addition of water, chemicals, or cheap sweeteners affect honey quality properties and pose health risks. This work presents a spot-test paper-based analytical device (PAD) for the colorimetric quantification of reducing sugars in honey, expressed as the sum of fructose and glucose, which was determined using Benedict’s reaction. Images of the spot-test PAD are captured with a smartphone camera under controlled light conditions and processed with the free web application Trigit. The increase in the color intensity in the blue channel gives a linear correlation with the concentration of reducing sugars in the range of 50–400 mg/L. The obtained limit of detection and quantification values were 11 mg/L and 37 mg/L, respectively. The proposed spot-test PAD offers a low-cost, biodegradable, and easily portable material for the rapid and simple quantification of reducing sugars in honey samples. Likewise, using the smartphone as a digitization system enables rapid data acquisition and analysis, making the method useful as a practical screening tool in food safety and quality control in small or non-centralized laboratories.

## 1. Introduction

Paper-based analytical devices (PADs) have become increasingly interesting for their application as low-cost, portable sensing platforms within analytical chemistry. Since their introduction in 2007 by Whitesides and co-workers [1], PADs have attracted considerable attention due to their simplicity, minimal reagent consumption, biodegradability, and suitability for decentralized analysis. Their design is mainly based on patterning hydrophilic zones onto cellulose substrates, which are typically delimited by hydrophobic barriers created through techniques such as photolithography [2], screen printing [3], inkjet printing [3], plasma oxidation [4], or wax printing [5]. Among them, wax printing is still one of the most widely used because it is rapid, inexpensive, and compatible with mass fabrication. Following this strategy, wax is printed onto cellulose paper following a digitally designed pattern and subsequently melted to penetrate the full thickness of the substrate, forming well-defined hydrophobic boundaries that confine liquid samples within hydrophilic test zones. Although this approach became the dominant strategy for large-scale PAD fabrication, it is noteworthy that commercial wax printers and their consumables were discontinued by manufacturers. In this context, alternative strategies have been explored, such as the pen-plotting technique [6]. 

PADs are particularly well suited for colorimetric detection strategies, which remain the most commonly used approach in these devices [7,8]. Colorimetric assays rely on the generation of visually detectable color changes in the presence of the analyte, enabling qualitative, semi-quantitative, or quantitative measurements. Qualitative results can often be interpreted by the naked eye, whereas quantitative analysis typically requires digitalization of the color intensity using smartphone cameras, digital cameras, or flatbed scanners [9,10,11]. The resulting images are then processed with dedicated image analysis software or web and mobile applications such as ImageJ [12], Trigit [13], or RGB Color Detector, among others, to acquire the color numerical information. Several color spaces can be used for obtaining the information contained in the captured image [14], including RGB (Red–Green–Blue), CMYK (Cyan–Magenta–Yellow–Black), HSV (Hue–Saturation–Value), CIELAB, and greyscale. Among them, the RGB color space is the most widely employed in colorimetric assays based on PADs. In this color space, each pixel is described by three numerical values ranging from 0 to 255, corresponding to the intensities of the red (R), green (G), and blue (B) channels. The variation in one or more of these channels can be directly correlated with the concentration of the target analyte. 

The integration of PADs with smartphone-based detection has opened new opportunities for point-of-need analysis, particularly in resource-limited settings where conventional laboratory instrumentation is not accessible [15,16]. Recent research has demonstrated that smartphone-assisted colorimetric analysis can achieve high analytical reliability when illumination, imaging parameters, and color space selection are properly controlled. Several studies have reported optimized workflows for quantitative smartphone-based sensing on PADs, emphasizing the importance of standardized lighting conditions, device calibration, and robust image processing pipelines [17,18,19]. These advances highlight the growing maturity of smartphone-enabled colorimetry and further support its use as a reliable readout strategy in PAD-based assays. One area where such portable and low-cost analytical tools are especially valuable is food quality control. In this context, the determination of sugars in foods and beverages is a key parameter for assessing nutritional value, authenticity, and compliance with regulatory standards. Among sugars, reducing sugars, mainly monosaccharides such as glucose and fructose, as well as certain disaccharides and glucose, are of particular interest due to their relevance in food processing. Excessive consumption of sugars has been associated with adverse health effects, including overweight, obesity, diabetes, cardiovascular diseases, cognitive decline, and even increased cancer risk. Consequently, accurate monitoring of sugar content in foods is essential both for consumer protection and for regulatory enforcement.

Honey represents a particularly challenging matrix in this context. It is one of the most adulterated food products worldwide, largely due to its high commercial value and complex composition. The major constituents of honey are the reducing sugars fructose and glucose, which together account for approximately 90–95% of its total sugar content. Fraudulent practices include dilution with water that accelerates fermentation, the addition of chemicals hindering its dietary properties, and the incorporation of low-cost sweeteners, which are the most widespread forms of adulteration of this food product. Ensuring the authenticity of honey is therefore critical for maintaining consumer trust, preserving its nutritional properties, and preventing health risks associated with adulterants. Regulatory frameworks such as the European Directive 2001/110/EC [20] and the Codex Alimentarius [21] establish minimum acceptable levels of reducing sugars, i.e., at least 60 g per 100 g for blossom honeys and at least 45 g per 100 g for honeydew honeys or their mixtures.

A wide range of analytical techniques has been developed for the determination of reducing sugars in honey. Chromatographic methods, including liquid chromatography with refractive index detection (LC-RI) [22], high-performance liquid chromatography with diode array detection (HPLC-DAD) [23], evaporative light scattering detection (HPLC-ELSD) [24], or mass spectrometry (SFC-MS, HPAEC-MS, and MALDI-TOF/MS) [25,26,27] offer high sensitivity and selectivity and allow differentiation between individual sugars. However, these methods require expensive instrumentation, trained personnel, and laboratory-based workflows, and they often involve long analysis times. Spectrophotometric and spectroscopic approaches, such as UV–Vis detection [28,29], visible and near-infrared spectroscopy (Vis-NIR) [30], and infrared spectroscopy (IR) [31], have also been explored as simpler alternatives. These methods can provide rapid, non-destructive analysis and are often combined with chemometric tools to extract meaningful information from complex spectral data. Nevertheless, their sensitivity may be lower than that of chromatographic techniques, and they may require calibration models that are specific to the type of honey analyzed. Furthermore, smartphone-based enzymatic methods [32] and microfluidic paper-based analytical devices (µPADs) [33] incorporating enzymes, nanoparticles, or metal–organic frameworks have been proposed for sugar analysis in honey. Nevertheless, these systems may still require specialized reagents, multistep fabrication, or calibration models tailored to specific honey types, highlighting the need for simpler, low-cost, and instrument-free alternatives.

In addition, conventional colorimetric reactions, such as Fehling’s reaction and Benedict‘s reaction, have long been used for the detection of reducing sugars due to their simplicity and low cost [34]. Fehling’s reagent must be freshly prepared from two separate components before each analysis, as the mixed reagent deteriorates rapidly. This instability limits its practicality and affects analytical reproducibility. The Benedict reagent, by contrast, is a single, stable mixture that can be stored for months, making it more convenient and safer to handle. Benedict’s reaction produces a color change that varies from blue to green, yellow, or red, depending on the concentration of reducing sugars, and is usually used for qualitative or semi-quantitative detection. However, it can also be adapted for quantitative analysis when combined with spectrophotometric or digital image-based detection, making it well suited for integration into PADs.

Despite the progress made in developing analytical methods for reducing sugars in honey, there remains a need for simple, low-cost, and portable approaches that can be used for rapid screening outside laboratory environments. The present work addresses the development of a spot-test PAD for the colorimetric quantification of total reducing sugars in honey using Benedict’s reaction. The device is fabricated using wax printing to create hydrophobic barriers and incorporates a simple workflow compatible with smartphone-based image acquisition. The analytical performance of the PAD, including linear range, limit of detection, limit of quantification, and applicability to real honey samples, is systematically evaluated. The results demonstrate that the proposed PAD provides a rapid and low-cost method suitable for routine screening of honey quality and authenticity in non-centralized laboratories. By combining a stable colorimetric reaction with digital image analysis, this approach contributes to the development of accessible sensing technologies for food safety monitoring and supports the broader adoption of PADs in decentralized analytical applications that rely on basic laboratory equipment.

## 2. Materials and Methods

### 2.1. Reagents and Solutions

All reagents were of analytical grade and used without further purification. Copper (II) sulfate pentahydrate (CuSO_4_·5H_2_O) was obtained from Probus (Badalona, Spain). Sodium citrate tribasic dihydrate (C_6_H_5_Na_3_O_7_·2H_2_O, 99%) was purchased from Sigma-Aldrich (Overijse, Belgium). Anhydrous sodium carbonate (Na_2_CO_3_, 99.5%) and sodium hydroxide (NaOH, 99%) were obtained from VWR Chemicals (Fontenay-sous-Bois, France). D-glucose monohydrate (C_6_H_12_O_6_·H_2_O, 97.5%) was supplied by Panreac (Barcelona, Spain) and D-fructose (C_6_H_12_O_6_, 99%) was purchased from Sigma-Aldrich (St. Louis, MO, USA). The ultrapure water (resistivity 18.2 MΩ·cm^−1^) was produced using a Millipore Simplicity purification system (Darmstadt, Germany).

The Benedict reagent was prepared by dissolving copper (II) sulfate (75 mM), sodium carbonate (2 M), sodium citrate (1.3 M), and sodium hydroxide (0.3 M) in ultrapure water. All components were mixed until complete dissolution, and the solution was stored at room temperature until use. Stock solutions (1000 mg/L) of glucose and fructose were prepared in ultrapure water and stored under refrigeration until use.

### 2.2. Instrumentation and Materials

Spot-test PAD fabrication was tested using commercial cellulose filter papers, Whatman™ grade Nos. 1, 540, 541, 542, and 602H (Cytiva, Munich, Germany). Hydrophobic barriers were printed as square-shaped patterns using a Xerox ColorQube 8580 wax printer (Kuala Lumpur, Malaysia) and heated using a forced-air oven (OVF, 30 L, 230 V, 50 Hz; Labbox, Spain). Colorimetric assays were performed using a hot-plate stirrer (Agimatic-N, Abrera, Spain) for reagent heating.

A portable cold white LED lightbox (PULUZ photo studio, Shenzhen, China) was used to ensure controlled illumination during image acquisition. A Moulinex Capriccio 1200 hair dryer was used for drying steps when required. A Sartorius analytical balance (model CP 124S) and a Sartorius microbalance (model MC 5), both from Göttingen (Germany), were used for weighing. 

Images of the PADs were captured using smartphone cameras from three different models: Xiaomi 11T, Samsung S8, and Samsung Galaxy Note 10+. 

### 2.3. Data Processing

The smartphone-based applications RGB Color Detector (The Programmer, v215.0.0, Google Play Store) and Color Analyzer (Kamusoft, v3.0.5, Google Play Store) were employed to directly process the images captured with the smartphone camera. In addition, the free software ImageJ v.1.54r and the free web application Trigit were also used for image processing and quantitative analysis. 

### 2.4. Design and Fabrication of Spot-Test PAD

Optimized spot-test PADs were fabricated using Whatman™ grade No. 1 cellulose filter paper sheet as the hydrophilic substrate. A pattern consisting of 5 × 5 mm black squares (line thickness: 1 pt) was printed onto the paper using a wax printer. The printed sheet was then heated in a forced-air oven at 130 °C for 30 s, allowing the wax to melt and permeate the full thickness of the paper. This process generated hydrophobic barriers (1 mm width) that defined the reaction/detection zones.

### 2.5. Honey Samples

Commercial honey samples were purchased from local supermarkets, including products from different brands and botanical origins. Multi-blossom honey was obtained from Granja San Francisco (origin: Pampeana region, Argentina). Monofloral honeys of orange blossom, heather, eucalyptus, lavender, rosemary, and thyme were acquired from Mellarius (origin: Hoces del Cabriel Natural Park, Spain). Honeydew and rosemary honeys were purchased from Mielso S.A. (origin: Almazora, Spain).

Additionally, three artisanal honeydew honeys were obtained from local producers: honeydew-1 from Ribeira Sacra (Ourense, Spain), honeydew-2 from Taboadelo (Pontevedra, Spain), and honeydew-3 of unknown origin.

Before analysis, honey samples were homogenized and diluted in ultrapure water to obtain working solutions suitable for colorimetric measurement. Dilution factors were selected to ensure that the reducing sugar concentration fell within the linear range of the method.

### 2.6. Experimental Procedure for the Determination of Total Reducing Sugars in Honey

First, 6 μL of Benedict reagent was added to each reaction zone of the spot-test PAD. Subsequently, 8 μL of either standard solution, blank, or honey sample was added. To prevent liquid spattering during heating, the spot-test PAD was completely dried before the colorimetric reaction. The Benedict reagent/standard, blank, or sample mixture was dried with a hot air dryer for 2 min (at around 65 °C), producing a dry spot. The spot-test PAD was then placed on a preheated hot plate at 110 °C for 90 s to perform the detection reaction. During heating, the appearance of a green coloration indicated the formation of the copper(I) product associated with the presence of reducing sugars. After heating, the PAD was removed from the hot plate and immediately placed inside a Puluz LED lightbox, which provides uniform and enclosed illumination, ensuring controlled illumination during image acquisition. The color developed in each reaction zone was digitized using the camera of a smartphone configured to ISO 500, exposure value (EV) +1, and white balance (WB) 5400 K. Each captured image of the detection zones was processed with the free web application Trigit [35], extracting the numerical values of the blue channel from the RGB color space. The analytical response was expressed as the mean color intensity difference ΔIc = blank − standard/sample. A schematic illustration of the PAD preparation and reaction steps is provided in Figure S1.

## 3. Results and Discussion

### 3.1. Preliminary Studies

Initial experiments were conducted to evaluate the behavior of Benedict’s reaction in the spot-test PAD. When the reaction mixture, i.e., the Benedict reagent and sugar standard, was transferred to the cellulose substrate, color development at room temperature was extremely slow, requiring approximately 24 h to become visible. This demonstrated the need for an external heat source to accelerate the reaction on the spot-test PAD. Several heating methods were evaluated, including forced-air and natural-convection ovens, microwave irradiation, infrared lamps, and a hot plate. The hot plate provided the fastest and most homogeneous color development, producing a uniform green coloration across the reaction zone within 2 min. Consequently, the hot plate was selected as the heating method for all subsequent experiments.

Color quantification was performed using the smartphone camera and processed with the free web application Trigit. Analysis of the RGB color space revealed that the blue channel (B) provided the highest analytical response for both fructose and glucose and was therefore selected for quantitative evaluation (Figure S2).

### 3.2. Optimization of Experimental Variables

A univariate optimization strategy was applied to identify the optimal conditions for the colorimetric determination of reducing sugars on the spot-test PAD. All experiments were performed using 500 mg/L standards of fructose and glucose. 

#### 3.2.1. Benedict Reagent: Concentration of Copper (II) Sulfate and Mass of Dried Reagents

**Figure 1:**
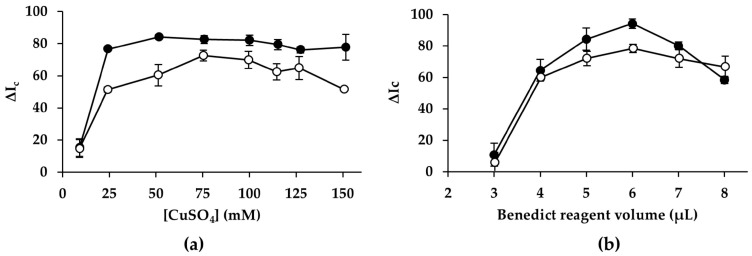
(**a**) Effect of CuSO_4_ concentration; (**b**) effect of Benedict reagent volume—mass of dried reagents (fructose data area shown as white markers and glucose data as black markers).

The concentration of copper (II) is critical for the formation of Cu_2_O and the resulting color intensity generation. To test the effect of copper (II) concentration, Benedict reagent was prepared with copper (II) sulfate concentrations ranging from 10 to 150 mM, while keeping sodium carbonate (2 M), sodium hydroxide (0.3 M), and sodium citrate (1.3 M) constant. As shown in Figure 1a, for copper (II) sulfate concentration below 25 mM, the analytical response was markedly reduced for both glucose and fructose. Under these conditions, the reaction is copper-limited, resulting in weak color development and poor sensitivity. By increasing the copper (II) sulfate concentration, the analytical response improved for both analytes. Under alkaline conditions, both fructose and glucose undergo base-catalyzed keto–enol tautomerization to form reactive enodiolate intermediates capable of reducing copper (II) to Cu_2_O. Fructose forms these intermediates more rapidly and efficiently than glucose, which first undergoes an aldose–ketose isomerization before enodiolization occurs [36,37]. As a result, fructose response reached a plateau at a concentration of 50 mM copper (II) sulfate, whereas glucose requires a higher copper (II) concentration, showing a maximum at 75 mM. To enable simultaneous determination of both monosaccharides, 75 mM copper (II) sulfate was selected as the optimal concentration for further studies.

Furthermore, to determine the optimal amount of Benedict reagent deposited on the PAD, volumes between 3 µL and 8 µL were tested. It was observed that volumes below 3 µL did not fully cover the reaction zone, and thus smaller volumes were not tested. As shown in Figure 1b, the analytical response for both monosaccharides rises as the volume of Benedict reagent added increases. The greater the amount of Benedict reagent, the higher the color intensity generated, resulting in better sensitivity and an increase in the analytical response until reaching a maximum at a volume of 6 µL. When larger volumes were deposited, a slight decrease in analytical response was observed. This behavior is attributed to the high concentration of carbonate and citrate salts present in the reagent, which, upon drying, produce a thicker crystalline layer on the paper surface. The formation of this solid film reduces the homogeneity of the Cu_2_O precipitate and interferes with color uniformity, ultimately diminishing the measurable color intensity. Based on these results, a volume of 6 µL of Benedict reagent (mass of dried reagent: 30 µg copper (II), 680 µg sodium carbonate, and 1520 µg sodium citrate) was selected for further experiments.

#### 3.2.2. Spot-Test PAD Size and Type of Cellulose Substrate

Optimization of the reaction/detection zone size is essential to achieve high analytical sensitivity and precision in paper-based assays. To evaluate the influence of spot dimensions on color development, square reaction zones of different sizes, i.e., 4 × 4, 5 × 5, 6 × 6, 7 × 7, and 8 × 8 mm, were patterned on the cellulose substrate. Into each zone, 6 µL of Benedict reagent and 5 µL of monosaccharide standard or blank solution were deposited.

**Figure 2:**
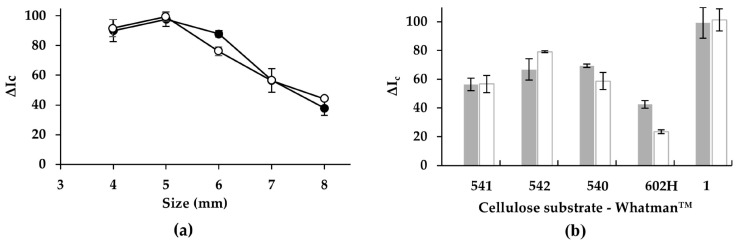
(**a**) Effect of spot-test PAD size; (**b**) effect of cellulose substrate type (fructose data area shown as white markers and glucose data as black markers).

Smaller reaction areas were expected to enhance sensitivity by concentrating the color produced within a reduced surface area, thereby increasing the apparent color intensity. The experimental results (Figure 2a) showed that the 5 × 5 mm size provided the highest analytical response for both glucose and fructose. When the reaction zone was reduced to 4 × 4 mm, the dried reagent accumulated predominantly along the perimeter of the zone, and small splattering effects were observed during heating on the hot plate. These phenomena compromised color uniformity and led to poorer analytical performance. Conversely, reaction zones larger than 5 × 5 mm yielded lower signal intensities. This decrease can be attributed to the effective dilution of the chromogenic Cu_2_O precipitate across a larger white cellulose background, which attenuates the recorded color intensity and reduces sensitivity. Based on these observations, a 5 × 5 mm reaction/detection area was selected as the optimal size for subsequent experiments with both monosaccharides.

In addition, the selection of the type of cellulose substrate is a critical parameter in the performance of PADs. Commercially available papers differ in pore size, thickness, and overall microstructure, which are physical characteristics that can significantly influence fluid transport, reagent distribution, and ultimately the analytical response. Table S1 summarizes the main properties of the cellulose substrates evaluated in this work.

Cellulose substrates with larger pore sizes are expected to facilitate faster diffusion and more uniform spreading of the reagent and sample within the reaction/detection zone, potentially improving the homogeneity of the color generated [38]. Furthermore, the thickness of the cellulose substrate can affect the apparent color intensity [39]; if the dried reagent and sample penetrate deeply into a thick, opaque substrate, the chromogenic Cu_2_O generated may be distributed through a larger volume, reducing the color intensity visible at the surface of the PAD. Therefore, the optimal substrate should provide an appropriate balance between pore size and thickness, ensuring efficient reagent distribution while maintaining durable and uniform color development.

As shown in Figure 2b, Whatman grade No. 1 yielded the best analytical performance for both glucose and fructose. Although Whatman 541 exhibits the largest pore size and the lowest thickness among the tested substrates, its reduced thickness appears to limit its capacity to retain the deposited reagent volume. During heating on the hot plate, this resulted in splattering and irregular spreading of the reaction mixture, compromising color uniformity and decreasing analytical sensitivity. A similar behavior was observed for Whatman 540 and Whatman 542, which also showed poor color homogeneity under the same heating conditions. In contrast, Whatman 602H, which has the smallest pore size, produced less uniform drying patterns and poorer analytical responses. The restricted porosity likely hindered adequate distribution of the reagent within the reaction zone, leading to uneven color formation.

Thus, Whatman grade No. 1 was selected as the cellulose substrate for the fabrication of the spot-test PADs used in this work, offering the best compromise between pore size, thickness, and colorimetric performance.

#### 3.2.3. Sample Volume

**Figure 3:**
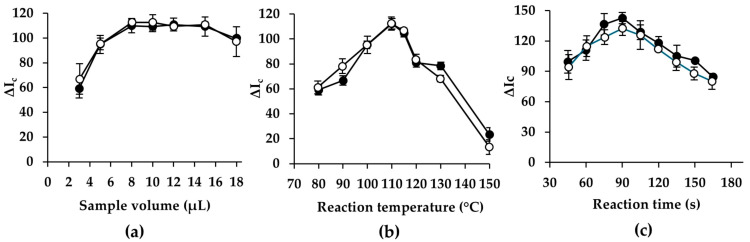
(**a**) Effect of sample volume; (**b**) effect of reaction temperature; and (**c**) effect of reaction time (fructose data area shown as white markers and glucose data as black markers).

The volume of sample deposited onto the reaction/detection zone of the spot-test PAD influences the intensity and uniformity of the colorimetric response. Increasing the sample volume raises the total amount of analyte delivered to the reaction zone, and thus a greater quantity of chromogenic product (Cu_2_O) is expected to form, providing higher color intensity and improved analytical sensitivity. To evaluate the influence of this parameter, sample/standard volumes in the range of 3–15 µL were tested. The obtained results, shown in Figure 3a, indicate that the analytical response increases progressively with sample volume, reaching a maximum for volumes between 8 and 14 µL for both glucose and fructose. This behavior is consistent with the expected increase in analyte mass contributing to the redox reaction.

However, practical considerations must also be considered. Before heating, the PAD must be allowed to dry completely to ensure reproducible color development. Larger sample volumes extend the drying time, thereby increasing the total analysis time and reducing the achievable sampling frequency. Beyond a certain point, the additional analyte provided by larger volumes does not translate into a proportionally greater analytical response, making the extra drying time useless. Considering both analytical performance and operational feasibility, a sample volume of 8 µL was selected as the optimal compromise. This volume provides a suitable and reproducible analytical response while minimizing drying time and enabling a higher throughput in routine analyses.

#### 3.2.4. Temperature and Reaction Time

Preliminary experiments demonstrated that Benedict’s reaction for both glucose and fructose proceeded extremely slowly on the cellulose substrate at room temperature, requiring more than 24 h before any visible color change can be observed. To accelerate the reaction and enable practical analytical performance, the spot-test PAD was heated directly on a hot plate, reducing the reaction time. Given the critical role of heating in promoting the redox reaction and hence the resulting color intensity, the effect of reaction temperature was carefully evaluated. Temperatures in the range of 80–150 °C were tested (Figure 3b). Temperatures above 150 °C caused visible burning of the cellulose substrate, making them unsuitable for spot-test PAD operation, so higher temperatures were discarded. Within the temperature range tested, the strongest and most reproducible color development was obtained at 110 °C, which produced high signal intensity with minimal standard deviation for both monosaccharides. Above 115 °C, even small fluctuations in temperature led to a marked decrease in analytical response and poorer precision. This sensitivity to temperature variations at higher settings may be attributed to rapid solvent evaporation and localized overheating, which can disrupt the uniform distribution of reagents, hinder consistent Cu_2_O formation, and influence color development, thus effecting the analytical response. A reaction temperature of 110 °C was selected as the optimal heating temperature for subsequent experiments, providing a robust balance between reaction speed, color intensity, and reproducibility.

In addition to temperature, the period of heating on the hot plate is key in determining the extent of copper (II) reduction by the monosaccharides and the resulting color intensity. To establish the optimal reaction time, the evolution of the colorimetric signal was evaluated over a range of 45–165 s at the previously selected temperature. The results, presented in Figure 3c, show that the analytical response increases progressively with heating time, reflecting the continued formation of colored product as the reaction proceeds. For both glucose and fructose, the signal reached a maximum at 90 s, after which no further improvement was observed. This plateau indicates that the reaction is essentially complete at this time, and additional heating does not enhance color development. Finally, a reaction time of 90 s was selected as optimal, providing strong and reproducible color formation while shortening the analytical procedure.

#### 3.2.5. Digitization Parameters: ISO, EV, and WB

When using smartphones that allow manual camera mode, three key parameters can be adjusted to control image acquisition, i.e., ISO (sensor sensitivity), exposure value (EV), and white balance (WB) [40]. The ISO setting determines the sensitivity of the camera sensor to light; higher ISO values increase sensitivity and allow image capture under lower illumination, but to the detriment of increased image noise. On the contrary, low ISO values require stronger illumination but yield cleaner images with reduced noise. The EV parameter regulates the exposure of the captured image by controlling the amount of white light reaching the sensor, thereby influencing the brightness and saturation of the recorded color. Finally, the WB setting adjusts the color temperature of the image, shifting the tonal balance toward warmer or cooler hues depending on the selected value.

**Figure 4:**
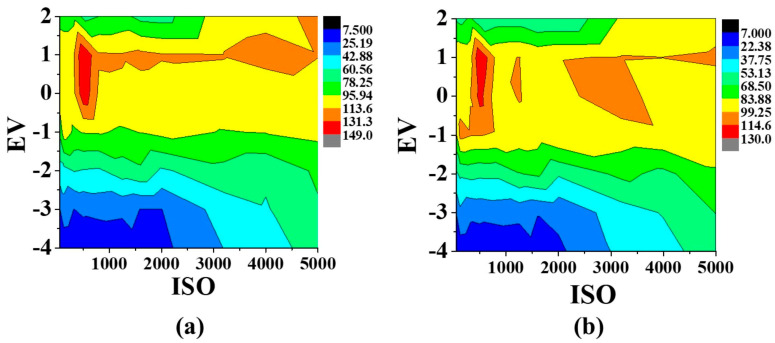
Effect of ISO and EV digitization parameters for (**a**) fructose and (**b**) glucose.

To find the optimal digitization conditions, a combined optimization of ISO and EV was first performed. Multiple images of the spot-test PAD were acquired at a fixed ISO value while systematically varying the EV setting in the range from +2 to −4. The resulting contour plots (Figure 4) revealed that, for both glucose and fructose, the optimal ISO value was 500, while the EV setting could range between 0 and +1 without compromising performance. To maximize color intensity while maintaining reproducibility, EV = +1 was selected.

Once ISO and EV were fixed at their optimal values (ISO = 500, EV = +1), the WB parameter was evaluated. As shown in Figure S1, the analytical signal increased with WB until reaching a plateau at approximately 5000 K for both monosaccharides. To ensure robust performance under typical ambient lighting conditions, a slightly higher value of 5400 K was chosen as a compromise, providing stable and reproducible color capture across all measurements.

The following optimized digitization parameters, ISO = 500, EV = +1, and WB = 5400 K, were used for all subsequent spot-test PAD image acquisitions to ensure consistent and high-quality analytical results.

### 3.3. Comparison of Devices and Image-Processing Tools for Total Monosaccharide Quantification

As established during the optimization of experimental parameters, image acquisition using a smartphone camera was selected as the digitization method for quantitative analysis of the spot-test PAD. Smartphones offer several advantages for decentralized analytical applications, including widespread availability, portability, and integrated high-resolution cameras. However, differences in camera hardware, sensor characteristics, and internal image processing algorithms across devices may influence the recorded color intensity and, consequently, the analytical results. To evaluate whether such differences are significant, the total monosaccharide content was quantified using three commercially available smartphones from different manufacturers, i.e., Xiaomi 11T, Samsung S8, and Samsung Galaxy Note 10+. All measurements were performed under optimized experimental conditions using a mixed glucose–fructose standard (300 mg/L).

The results obtained with the three devices are summarized in Table 1. A one-way analysis of variance (ANOVA) with equal sample size (n = 3) was applied to assess potential differences among devices (detailed information in the Supplementary Materials). Prior to the ANOVA, Bartlett’s test confirmed the homogeneity of variances ($c_{0}^{2}$ < $c_{c}^{2}$). The ANOVA results showed that F_0_ < F_tab_ supports the null hypothesis (H_0_) and demonstrates that no statistically significant differences exist among the results obtained with the three smartphones. These findings indicate that, under controlled acquisition conditions, the proposed method is robust with respect to the choice of smartphone device.

In addition, the influence of using different image processing applications on the quantification of total monosaccharides was evaluated using the Xiaomi 11T smartphone. Four freely accessible tools were tested: the mobile apps RGB Color Detector and Color Analyzer, the web-based platform Trigit, and the open-source image analysis software ImageJ. A real honey sample (thyme honey) with a known monosaccharide content was analyzed under optimized conditions. As before, Bartlett’s test confirmed the homogeneity of variances, and subsequent ANOVA analysis showed F_0_ < F_tab_, indicating that no significant differences were observed among the results obtained with the different applications for image processing (Table S2). Moreover, the experimental monosaccharide percentages obtained with each image processing tool were compared with the reference value provided by the manufacturer using a two-tailed Student’s t-test (α = 0.05). In all cases, t_cal_ < t_tab_, confirming that the experimental values did not differ significantly from the certified reference value (Table S2).

These results demonstrate that the spot-test PAD-based method developed for total monosaccharide quantification is highly robust, showing no significant dependence on the smartphone model or the image processing tool employed. This robustness reinforces the suitability of the method for decentralized, low-cost analytical applications where users may rely on different devices and freely available software.

### 3.4. Analytical Characteristics

**Figure 5:**
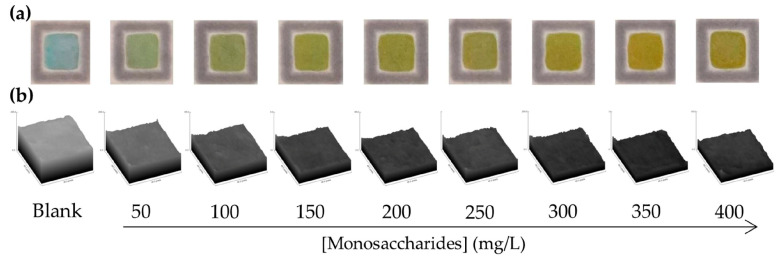
(**a**) Representative spot-test PAD images for each calibration standard (0–400 mg/L total monosaccharides) and (**b**) ImageJ surface plot profiles.

Once the optimal experimental conditions were established, the analytical performance of the proposed PAD-based procedure was evaluated. A calibration curve was constructed using standards based on the total monosaccharide content, where each calibration point corresponds to the sum of the glucose and fructose concentrations. The method exhibits an equivalent response for both monosaccharides under the optimized conditions. The resulting calibration curve (Figure S4) is described by the following linear regression equation:

*∆I_c_* = 0.1714 (±0.0025) [*monosaccharides*] mg/L + 22.4692 (±0.6400) (1)

where ΔI_C_ represents the increase in color intensity (blank intensity minus sample or standard intensity). The method shows excellent linearity up to 400 mg/L, with a correlation coefficient of r = 0.9993, indicating a highly reliable linear response within this concentration range. Representative images of the spot-test PAD for each calibration standard and blank, along with their corresponding surface plot profiles (Figure 5), confirm the homogeneity of the color development across the detection zone.

The limit of detection (LOD) and limit of quantification (LOQ) were calculated from the standard error of the intercept (a) and the slope (b) of the calibration curve using the conventional expressions LOD = 3a/b and LOQ = 10a/b. The calculated values were LOD = 11.2 mg/L and LOQ = 37.3 mg/L, demonstrating that the method is sufficiently sensitive for the determination of monosaccharides in real samples such as honey. Method repeatability was assessed at two concentration levels (100 and 300 mg/L), expressed as relative standard deviation (RSD). The obtained RSD values were 8% and 6%, respectively, which are acceptable for paper-based colorimetric assays. Intermediate precision (between-day reproducibility) was also evaluated at 300 mg/L, yielding an RSD of 2%, confirming the robustness and stability of the method over time.

The analytical performance of the proposed PAD-based colorimetric method was compared with previously reported procedures for the determination of reducing sugars in honey (Table 1). It should be noted that the methods summarized in Table 1 differ in their analytical target, e.g., some quantify total reducing sugars and others determine individual monosaccharides. Therefore, the comparison is qualitative and intended to contextualize the range of existing analytical strategies. As summarized, most established methodologies rely on chromatographic or spectroscopic instrumentation [24,26,27,41], which, although they provide high sensitivity and selectivity, require specialized equipment, trained personnel, and relatively long analysis times. 

Colorimetric alternatives, such as the Mo-B particulate colorimetric system [28] and the Tollens-based UV–Vis method [29], have also been reported as simpler and more sensitive approaches. However, these procedures require microwave activation or spectrophotometric instrumentation, which limits their portability and makes them less suitable for decentralized analysis. 

Smartphone-based enzymatic assays [32] offer portability but typically exhibit higher LOQs. Beyond instrumental and solution-phase colorimetric methods, some micro-PADs have recently been proposed for the determination of glucose and fructose in honey [33]. However, these approaches typically rely on enzymatic reactions, nanoparticle formation, metal–organic frameworks, or electrochemical transduction, which would increase cost, fabrication complexity, or reagent instability. Simple reagent-based PADs for total sugar content determination remain scarce. In this context, the proposed spot-test PAD allows a sensitivity between that of the most sensitive spectrophotometric methods and the less sensitive smartphone-based enzymatic assays. Importantly, unlike chromatographic or spectroscopic techniques, the proposed method requires no instrumentation beyond a smartphone camera, uses inexpensive reagents, and enables rapid analysis with minimal sample preparation. Although it does not reach the ultra-low detection limits of advanced systems, its combination of low cost and adequate sensitivity makes it highly suitable for decentralized or field-based screening of total monosaccharide content in honey.

### 3.5. Application of the Proposed Method to the Analysis of Reducing Sugars in Honey Samples

The method was validated against the certified reference material, BCR-644. Portions of the material were accurately weighed according to the BCR-644 protocol: 5.000 g for sucrose + lactose determination and 2.200 g for glucose (as starch) determination, dried at 102 ± 1 °C to constant mass, and cooled in a desiccator prior to analysis. Free sugars (sucrose and lactose) were extracted by dispersing the dried sample in ultrapure water at room temperature for 1 h, swirling from time to time. After, it was quantitatively transferred to a 250 mL volumetric flask. The obtained solution was appropriately diluted with ultrapure water into the linear range before analysis. For glucose, enzymatic hydrolysis was applied based on the BCR-644 protocol. Briefly, starch was liquefied with thermostable α-amylase at 100 °C, followed by saccharification with amyloglucosidase after pH adjustment. The hydrolysate was quantitatively transferred to a 250 mL volumetric flask. Before glucose analysis, the hydrolysate was diluted with ultrapure water into the linear range. The PAD results obtained for sucrose + lactose and glucose were in good agreement with the certified values (Table 2).

The Student’s *t*-test was applied, fulfilling the conditions t_exp_ < t_crit_ (t_crit_ = 2.776 for N−1 = 4 freedom degrees). The results confirmed that non-significant differences (*p* = 0.05) were observed between the certified values and the found contents, showing that the spot-test PAD approach provides accurate quantification of reducing sugars and demonstrates its suitability for routine quality control applications.

Finally, the proposed approach was applied to the determination of total reducing sugars expressed as monosaccharides = fructose + glucose in twelve honey samples, i.e., nine commercial honeys, including multi-blossom, honeydew, and monofloral honeys (orange blossom, heather, eucalyptus, lavender, rosemary, and thyme), and three artisanal honeydew honeys. The results obtained are summarized in Table 3. Furthermore, representative PAD images for these samples are included in the Supplementary Materials (Figure S5). For the commercial multi-blossom, honeydew, and monofloral honeys, the percentage of total reducing sugars ranged from 72.6 to 77.5% m/m; in all cases, the values exceeded the minimum value established by the European Directive 2001/110/EC (≥60% m/m) [20]. These results confirm compliance with the regulatory requirement for reducing sugar content and do not indicate excessive water dilution, which would reduce the percentage of reducing sugars. However, this parameter alone cannot rule out adulteration modes involving the addition of reducing sugar syrups. The artisanal honeydew honey samples showed a reducing sugar content between 61.7 and 74.6% m/m, which also meets the regulatory requirement for honeydew honeys (≥45% m/m).

For the commercial honeys, Student’s t-test was performed to compare the experimental mean percentage of reducing sugars obtained with the PAD and the values determined using the AOAC Official Method 920.183, Lane–Eynon titration [48] (Table 3). In all cases, the calculated t_exp_ value was lower than the critical value (t_exp_ < t_crit_), demonstrating that no significant differences exist between the values obtained with the proposed method and the official reference method. For completeness, the monosaccharide values declared on product labels are also included in Table 3 as contextual information.

## 4. Conclusions

A spot-test PAD was developed and optimized for the rapid colorimetric determination of total monosaccharides, expressed as the sum of glucose and fructose, in honey using Benedict‘s reaction. The device enables copper (II) reduction to Cu_2_O directly on paper, producing a stable color signal proportional to the concentration of monosaccharides and quantifiable with a smartphone. The assay requires only 90 s, uses minimal reagent volumes, and can incorporate multiple detection zones to increase throughput.

The proposed method showed good accuracy when validated against the certified reference material, BCR-644, yielding results consistent with the certified values. Moreover, application to twelve honey samples demonstrated total reducing sugar contents between 61.7 and 77.5% m/m, with all samples meeting the requirements of Directive 2001/110/EC. For commercial honeys, no significant differences were observed between the experimental values and the sugar content declared on product labels.

The developed spot-test PAD provides a fast, low-cost approach for quantifying total monosaccharides in honey, requiring only basic laboratory equipment such as a hot plate to perform the colorimetric reaction. Its compatibility with smartphone-based detection streamlines data acquisition and analysis, making the method a practical screening tool for food quality and authenticity assessment in small or non-specialized laboratories prior to centralized analyses. 

## Figures and Tables

**Table 1:** Recent reported procedures for the determination of reducing sugars in honey samples (the methods listed target different analytes, i.e., total reducing sugars or individual monosaccharides, and report results in different units, as presented in the original publications; comparisons are therefore qualitative).

Method	LOD (mg/L)		LOQ (mg/L)		RSD (%)	Linear Range (mg/L)	Ref
	Gluc	Fruc	Gluc	Fruc			
HPLC-ELSD	0.16	0.11	0.49	0.33	0.1–0.5	40–2000	[24]
HPAEC-MS	0.006	0.02	0.02	0.06	3.0	0.05–4	[26]
MALDI-TOF/MS	1	2	n.a	n.a.	4.2–11.9	2–100	[27]
CZE-LIF	28.8	--	90.1	--	1.4–13.7	90–1800	[41]
UV–Vis based on the Mo-B particulate system	30	--	n.a.	--	8.02	50–1000	[28]
UV–Vis based on Tollen’s reagent (Ag NPs)	0.007	--	0.023	--	0.9	0.17–1.30	[29]
Colorimetric enzymatic NAD^+^/GDH-Fe(III) system	n.a.	--	900	--	5	900–7200	[32]
Enzymatic-based MOF chemiluminiscence	3.6	22.3	n.a	n.a	2.1–4.7	9–9000	[42]
CNTs-Ni(II)-2,3dhS modified electrode	2.16	2.88	6.48	8.64	7–9	LOQ–23.4	[43]
Electrochemical sensor based on a NiFe alloy	0.46		2.52		1.3–5.0	9–54	[44]
e-PAD based on cobalt phthalocyanine–ionic liquid–graphene composite	0.12	--	n.a.	--	1.8–900	4.6	[45]
Multi-enzyme cascade micro-PAD using CeO_2_@MOF system	9.0		30.6		2.4	18–180	[46]
CL paper device	5.4	7.2	n.a	n.a	3.4	18–1800	[47]
Colorimetric PAD based on Benedict’s reaction	11.2		37.3		6–8	LOQ–400	This work

HPLC-ELSD: High-performance liquid chromatography with evaporative light-scattering detection; HPAEC-MS: high-performance anion-exchange chromatography coupled to mass spectrometry; MALDI-TOF/MS: matrix-assisted laser desorption/ionization–time-of-flight mass spectrometry; CZE-LIF: capillary zone electrophoresis with laser-induced fluorescence detection; GDH: glucose dehydrogenase; MOF: metal–organic framework; CNTs: carbon nanotubes; PAD: paper-based analytical device; CL: chemiluminescence; LOD: detection limit; LOQ: quantification limit; and RSD: relative standard deviation.

**Table 2:** Determination of sugars in BCR-644 using the proposed spot-test PAD.

Sugar	Certified Value (g/100 g)	Found Value (g/100 g) n = 5	Recovery (%) n = 5	RSD (%) n = 5	t_crit_
Sucrose + lactose	32.05	31.7 ± 1.2	99.1 ± 3.7	3.8	−0.652
Glucose (as starch)	35.1 ± 1.2	33.8 ± 1.3	96.3 ± 3.9	4.0	−2.236

**Table 3:** Analytical results for total monosaccharide (as the sum of fructose and glucose) determination in honey samples.

	Honey Sample (Brand)	Total Monosaccharides (% m/m) ± s (n = 3)	AOAC 920.183 Official Method (% m/m) ± s (n = 3)	Labelled Monosaccharides Content (% m/m) ^1^	t_exp_ ^2^
Multifloral	Multifloral (Granja San Francisco)	78.3 ± 0.6	77.8 ± 0.4	79.0	1.4
	Honeydew (Mielso S.A.)	67.5 ± 0.8	68.7 ± 0.5	66.0	−2.6
	Honeydew-1 (artisanal)	74.6 ± 0.7	75.0 ± 0.3	--	−1.0
	Honeydew-2 (artisanal)	61.7 ± 3.0	63.7 ± 0.6	--	−1.1
	Honeydew-3 (artisanal)	67.0 ± 2.0	65.1 ± 1.0	--	1.7
Monofloral	Orange blossom (Mellarius)	72.6 ± 3.1	73.0 ± 0.7	72.0	−0.2
	Heather (Mellarius)	75.8 ± 1.3	76.3 ± 0.5	75.2	−0.7
	Eucalyptus (Mellarius)	77.5 ± 2.4	76.0 ± 0.3	75.2	1.1
	Lavender (Mellarius)	72.6 ± 3.1	73.1 ± 1.0	75.2	−0.3
	Rosemary (Mellarius)	76.1 ± 1.7	77.4 ± 0.8	75.2	−1.3
	Rosemary (Mielso, S.A.)	75.9 ± 1.8	76.3 ± 0.6	75.0	−0.4
	Thyme (Mellarius)	75.4 ± 1.7	75.1 ± 0.8	75.2	0.3

^1^ Values for total monosaccharide content as indicated on the labels of the commercial honey samples. ^2^ Student’s *t*-test; t_crit_ (*p* = 0.05, two-tailed, N − 1 freedom degrees = 2) = 4.303.

## Data Availability

The original contributions presented in this study are included in the article/Supplementary Material. Further inquiries can be directed to the corresponding author.

## Supplementary Materials

The following supporting information can be downloaded at: https://www.mdpi.com/article/10.3390/s26103031/s1, Figure S1. Step-by-step preparation and reaction workflow of the spot-test PAD; Figure S2. Effect of the different color spaces on the analytical response; Figure S3. Optimization of the white balance (WB) settings; Figure S4. Calibration curve; Figure S5. Representative images of PADs developed with the twelve honey samples analyzed in this study; Table S1. Pore sizes and thicknesses of the different cellulose substrate types used, as specified by the manufacturer; Table S2. Total monosaccharide concentrations obtained using various image processing tools; S1. ANOVA statistical analysis for smartphone and image processing tool comparison; S2. Student’s *t*-test for comparing the sample mean concentration with a reference value for commercial honey samples.

## Abbreviations

The following abbreviations are used in this manuscript:

ANOVA	Analysis of variance
CL	Chemiluminescence
CMYK	Cyan–Magenta–Yellow–Black
CNTs	Carbon nanotubes
CZE-LIF	Capillary zone electrophoresis with laser-induced fluorescence detection
EV	Exposure value
GDH	Glucose dehydrogenase
HPAEC-MS	High-performance anion-exchange chromatography coupled to mass spectrometry
HPLC-DAD	High-performance liquid chromatography with diode array detection
HPLC-ELSD	High-performance liquid chromatography with evaporative light scattering detection
HSV	Hue–Saturation–Value
IR	Infrared spectroscopy
ISO	Sensor sensitivity
LC-RI	Liquid chromatography with refractive index detection
LOD	Detection limit
LOQ	Quantification limit
MALDI-TOF/MS	Matrix-assisted laser desorption/ionization–time-of-flight mass spectrometry
MOF	Metal–organic framework
PAD	Paper-based analytical device
µPAD	Microfluidic paper-based analytical device
RGB	Red–Green–Blue
RSD	Relative standard deviation
SFC-MS	Supercritical fluid chromatography coupled to mass spectrometry
Vis-NIR	Visible and near-infrared spectroscopy
WB	White balance
