# Supplementary material for: Smartphone-Assisted Paper-Based Analytical Device for Rapid Colorimetric Detection of Total Reducing Sugars in Honey
Source: Sensors (Basel). 2026 May 11;26(10):3031. doi: 10.3390/s26103031 (PMC13210989; doi:10.3390/s26103031)
Supplement: Supplementary file 1 [file sensors-26-03031-s001.zip › sensors-4265195-supplementary.pdf]

SUPPLEMENTARY MATERIAL

Smartphone-assisted paper-based analytical device for rapid colorimetric detection of total reducing sugars in honey

Alicia Carro, Isela Lavilla, Carlos Bendicho, Vanesa Romero*

Centro de Investigación Mariña, Departamento de Química Analítica y Alimentaria, Grupo QA2, Edificio CC Experimentais, Universidade de Vigo, Campus de Vigo, As Lagoas Marcosende, 36310 Vigo, Spain;
* Correspondence: vromero@uvigo.gal (V. Romero)

Contents

Figure S1. Step-by-step preparation and reaction workflow of the spot-test PAD.

Figure S2. Effect of the different color spaces on the analytical response.

Table S1. Pore sizes and thicknesses of the different cellulose substrate types used, as specified by the manufacturer.

Figure S3. Optimization of the white balance (WB) settings.

Figure S4. Calibration curve.

Table S2. Total monosaccharide concentrations obtained using various image processing tools.

Figure S5. Representative images of PADs developed with the twelve honey samples analyzed in this study.

S1. ANOVA statistical analysis for smartphone and image processing tool comparison.

S2. Student's t-test for comparing the sample mean concentration with a reference value for commercial honey samples.

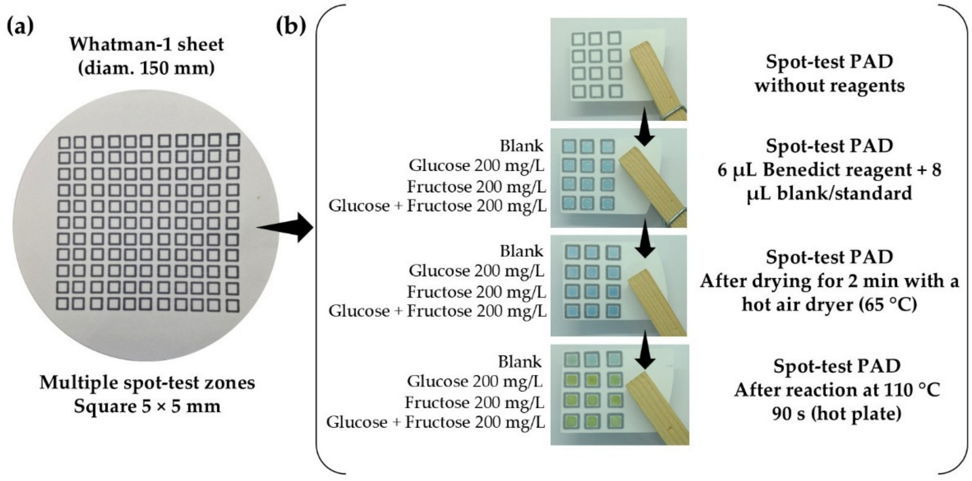

Figure S1. (a) Whatman-1 sheet containing multiple 5 × 5 mm reaction zones; (b) reaction workflow: spot-test PAD before reagent addition, spot-test PAD after addition of Benedict reagent and sample/standard/blank, spot-test PAD after drying for 2 min at around 65 °C, and spot-test PAD after the colorimetric reaction at 110 °C for 90 s. Includes representative images of blank, glucose, fructose, and glucose + fructose standards (200 mg/L).

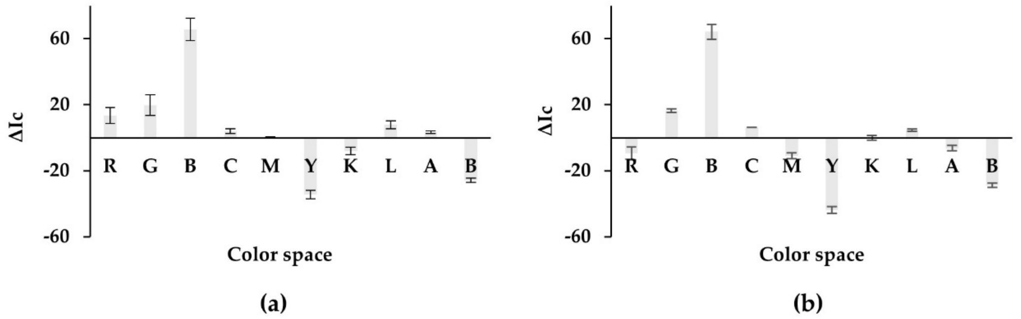

Figure S2. Effect of the different color spaces on the analytical response for **(a)** fructose and **(b)** glucose.

Table S1. Pore sizes and thicknesses of the different cellulose substrate types used, as specified by the manufacturer.

Type of Whatman™ substrate	Pore size (µm)	Thickness (µm)
541	20 – 25	155
542	2.7	150
540	8	160
602H	2	160
1	11	180

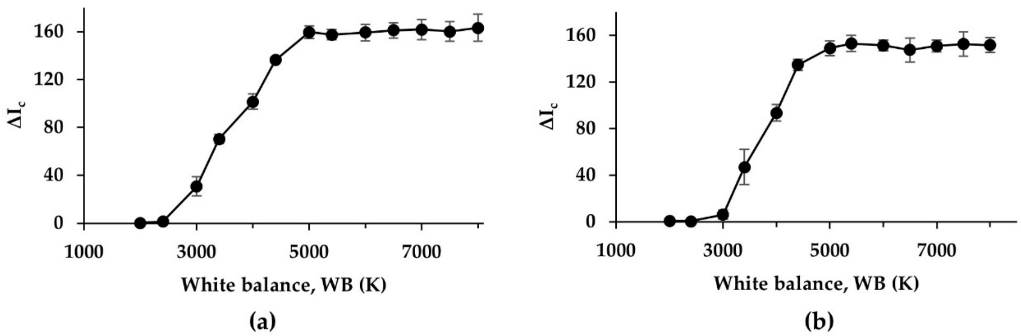

Figure S3. Optimization of the white balance (WB) settings: **(a)** fructose and **(b)** glucose.

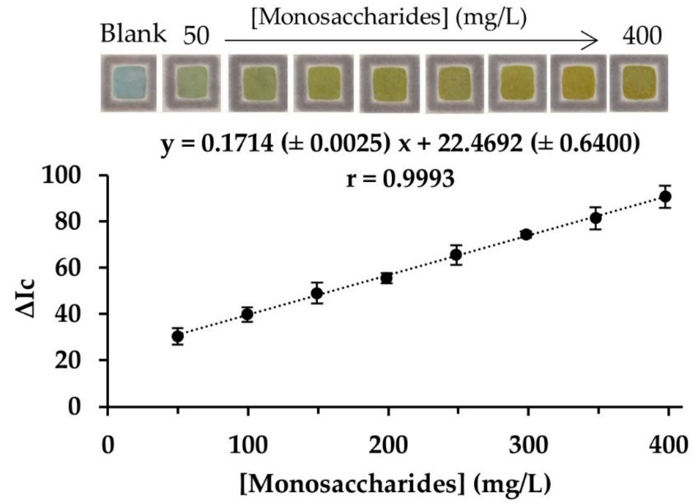

Figure S4. Calibration curve for the quantification of total monosaccharides (fructose + glucose).

Table S2. Total monosaccharide concentrations obtained using different image processing applications and software (reference sample: Mellarius thyme honey, [monosaccharides] = 75.2 % m/m).

Application/Program	[monosaccharides] % m/m	[1]t_{exp}
RGB Color Detector	75.4 ± 1.7 (n = 5)	0.263
Color Analyzer	78.1 ± 2.4 (n = 5)	1.907
Trigit	76.7 ± 1.9 (n = 5)	1.765
ImageJ	76.3 ± 1.4 (n = 5)	1.757

[1] Student's t-test; t_{crit} (p = 0.05, two-tailed, N – 1 freedom degrees = 4) = 2.776.

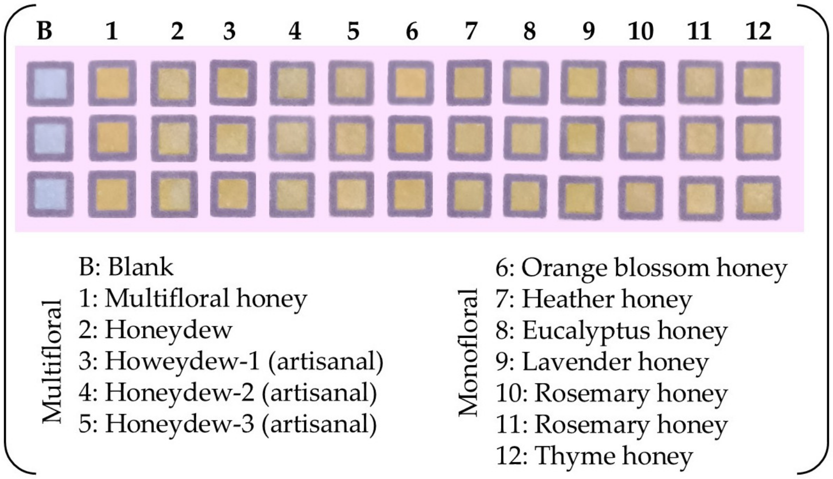

Figure S5. Representative images of PADs developed with the twelve honey samples analyzed in this study.

S1. ANOVA statistical analysis for smartphone and image processing tool comparison.

S1(a) Comparison of the results obtained using different smartphones.

Bartlett's test for homogeneity of variances

Group (i)	Data number (n_i)	Average ± standard deviation (s_i)	Variance (s_i^2)
1	3	252.6 ± 16	256
2	3	242.2 ± 8.4	70.6
3	3	222.8 ± 9.3	86.5

First, the pooled variance (s^2) is calculated using the following equation:

$$s^2 = \frac{(n_1 - 1)s_1^2 + (n_2 - 1)s_2^2 + (n_3 - 1)s_3^2}{(n_1 - 1) + (n_2 - 1) + (n_3 - 1)}$$

Using the experimental data, $s^2 = 137.7$.

Next, the test statistic is computed:

$$c_0^2 = \frac{1}{C}\left|(N - h)\ln s^2 - \sum_{i=1}^{h}(n_i - 1)\ln s_i^2\right|$$

$$C = \frac{1 + \sum_{i=1}^{h}\left(\frac{1}{n_i - 1} - \frac{1}{N - h}\right)}{3(h - 1)}$$

where N is the total data (in this case, $N = 9$); h is the number of groups (in this case, $h = 3$).

With the experimental data, $c_0^2 = 1.03$. On the other hand, the critical chi-square value (c_c^2) for $h - 1$ freedom degrees and $\alpha = 0.05$ is 5.99. $c_0^2 < c_c^2$. Therefore, the null hypothesis (H_0) is accepted, indicating that the variances are homogeneous.

One-way ANOVA test with an equal number of observations

Next, the one-factor ANOVA test is applied. First, the average of the within-series variances is calculated, $s_{res}^2 = 137.7$. Then, the variance of the means is computed, $s_m^2 = 228.8$. Finally, the F statistic is calculated:

$$F_0 = \frac{ns_m^2}{s_{res}^2} = \frac{3 \cdot 228.8}{137.7} = 4.985$$

The critical F value for a = 0.05 (one-tailed, $h - 1$ and $h \times (n - 1)$ freedom degrees), $F_{crit} = 5.143$. Since $F_0 < F_{crit}$, the null hypothesis (H_0) is accepted, indicating that there are no significant differences among the results of the different series.

S1(b) Comparison of the results obtained using different image processing tools.

Bartlett's test for homogeneity of variances

Group (i)	Data number (n_i)	average ± standard deviation (s_i)	Variance (s_i^2)
1	5	75.4 ± 1.7	2.9
2	5	78.1 ± 2.4	5.8
3	5	76.7 ± 1.9	3.6
4	5	76.3 ± 1.4	2.0

First, the pooled variance (s^2) is calculated using the following equation:

$$s^2 = \frac{(n_1 - 1)s_1^2 + (n_2 - 1)s_2^2 + (n_3 - 1)s_3^2}{(n_1 - 1) + (n_2 - 1) + (n_3 - 1)}$$

Using experimental data, $s^2 = 3.57$.

Next, the statistic is computed:

$$c_0^2 = \frac{1}{C}\left|(N - h)\ln s^2 - \sum_{i=1}^{h}(n_i - 1)\ln s_i^2\right|$$

$$C = \frac{1 + \sum_{i=1}^{h}\left(\frac{1}{n_i - 1} - \frac{1}{N - h}\right)}{3(h - 1)}$$

where N is the total data (in this case, $N = 20$); h is the number of groups (in this case, $h = 4$).

With the experimental data, $c_0^2 = 6.15$. On the other hand, the critical chi-square value (c_c^2) for $h - 1$ freedom degrees and $\alpha = 0.05$ is 7.81. $c_0^2 < c_c^2$. Therefore, the null hypothesis (H_0) is accepted, indicating that the variances are homogeneous.

One-way ANOVA test with an equal number of observations

Next, the one-factor ANOVA test is applied. First, the average of the within-series variances is calculated, $s_{res}^2 = 3.57$. Then, the variance of the means is computed, $s_m^2 = 2.2$. Finally, the F statistic is calculated:

$$F_0 = \frac{n s_m^2}{s_{res}^2} = \frac{5 \cdot 2.2}{3.57} = 3.077$$

The critical F value for $\alpha = 0.05$ (one-tailed, $h - 1$ and $h \times (n - 1)$ freedom degrees), $F_{crit} = 3.239$. Since $F_0 < F_{crit}$, the null hypothesis (H_0) is accepted, indicating that there are no significant differences among the results of the different series.

S2. Student's t-test for comparing the sample mean concentration with a reference value for commercial honey samples.

To compare the total monosaccharide concentrations found in the commercial honey samples with the concentrations indicated on the product labels (reference value), Student's t-test is applied. To calculate t_{exp} (reported in Table 4 of the main manuscript), the following equation is used:

$$t = \frac{(\bar{x} - \mu)\sqrt{n}}{s}$$

where $\bar{x}$ is the mean value of the measured concentration, s is the standard deviation of the measurements, n is the number of replicates, and μ is the reference value.
